# The Organic Index of Human Longivity

**Published:** 1863-11

**Authors:** W. Byrd Powell

**Affiliations:** Covington, Ky.


### CHICAGO MEDICAL EXAMINER.

N. S. DAVIS, M.D., EDITOR.

VOL. IV. NOVEMBER, 1863. NO. 11.

### Original Contributions.

### ARTICLE XXX.

### THE ORGANIC INDEX OF HUMAN LONGEVITY.

BY W. BYRD POWELL, M.D., of Covington, Ky.

MR. EDITOR:—Early in my practice, as a physician, my observations induced me to suspect that human longevity was not so referable to the incidental influences of life as was generally supposed. I observed that some people, who appeared to be soundly physiological and of temperate and prudent habits, died comparatively young, and of disease of comparatively moderate violence. That others lived to old age, with habitually imprudent and intemperate habits, and seemed greatly invulnerable to epidemic and other causes of disease; and, if assailed, recovered. The more I multiplied my observations the more I became impressed with the suspicion, that these different results had an organic cause. Being at this time engaged in repeating the observations of Dr. GALL, it became suggested to me as being possible, perhaps probable, that human longevity might be indicated by some cerebral development, and, henceforth, my observation was directed to the discovery of it.

Under this course of observation, it was not long before I was brought to the conviction, that vigorous life, or such as is attended by an energetic manifestation of the vital functions generally, is associated with a broad development of the cerebellum and of the base of the cerebrum; and, for the sake of distinction, I designated this class by the epithet of "vigorous life." But many years subsequently, Dr. MARSHALL HALL designated it by the more elegant epithet of "high stimulus." I do not remember that he indicated the organic index of this class. This class is particularly liable to acute forms of disease, as acute rheumatism, pneumonia pleuritis, &c., and a large proportion of this class die comparatively young, but a respectable minority live to old age; and the wherefore of this will be explained in the sequel.

Per contra, there is another class which is distinguished by the base of the cerebrum and the cerebellum being comparatively contracted. The vital functions of this class are never manifested vigorously, but frequently with much activity. This class generally manifests much tenacity of life. Many of this class are perpetually complaining of ill health and the expectation of an early death, but still live, and with wonderful tenacity of life, to the age of three score and ten years or older. I denominated this the vitally tenacious class, and Dr. HALL, by the epithet of "high dynamis," but neither is strictly correct, because a majority of those having a scrofulous diathesis belong to this class, and, generally, they do not live to be old. But those of this class who are exempt from any strumous taint have, usually, great tenacity of life, not unfrequently living a hundred years. The oldest individual I ever saw was of this class, and said to be one hundred and seventy years old, and the evidences of the fact, both physical and written, were to me conclusive of the fact. The individual was a negress, a native of Africa. The cornea of her eyes had a chalky opacity; her great toe nails projected beyond the toes three-fourths of an inch, and were half an inch thick, resembling the hoofs of an ass; her audition, however, was morbidly acute.

After making the preceding observations, about twenty-five years elapsed before I made any further note-worthy advance in this direction; but, in the meantime, I accumulated a cabinet of four hundred human crania, consisting of those of many peoples. Upon one occasion, when in my cabinet, it became suggested to me that as a broad development of the brain indicated vigorous life, that, possibly, the depth of the brain might indicate tenacious life or high dynamis, and immediately proceeded to ascertain how the fact might be. I took from my cases two crania: one was that of a white man who had died of phthisis, and the other that of a white man who, in the vigor of life and health, was executed for murder. To the former I extended a small line from the occipital protuberance to the anterior inferior lateral angle of the os frontis, and found the space between the line and the meatus auditorius to be two-eighths of an inch. I applied the line in like manner to the latter, and found the space between the line and the meatus auditorius to be one inch and three-eighths. As the two crania were, in the abstract, of about the same size, it appeared to me that the remarkable difference between the two in the depth of the brain's base was, probably, significant of some important truth, and, hence, I resolved upon a more methodical course.

I placed on my table the crania of five white men who had died, respectively, of phthisis, and measured them as above indicated, and divided the sum of their measures by five, and found the average to be three-sixteenths of an inch. I replaced these five crania by five others of white men who died by mechanical violence, and measured them in like manner, and found their average to be an inch and three-eighths. From these measures, though but few, I could not doubt that the depth of the brain's base was, probably an index of longevity, —high dynamis. I had in my cabinet the crania of four suicides, and found their average to be within an inconsiderable fraction of that of the consumptives, and have since observed that those having a suicidal diathesis measure about as do those having a consumptive diathesis, hence, I have been forced to regard suicide as a normal death. It is, therefore, preposterous to enquire why any one committed suicide, for, at most, nothing more can be learned than the exciting cause, which very frequently is some very trivial circumstance, just as a very inconsiderable exposure of the person may excite a scrofulous diathesis into consumption.

I continued this course of investigation in my cabinet, as opportunities offered, for several weeks, to satisfy myself on many of the relations of the subject as they became suggested to me. I selected the crania of those who were, as nearly as possible, exclusively of high stimulus: in these I found the cerebellum and the base of the cerebrum to be broadly developed, but had but little depth,—all of them had died young. Amongst these was that of a Polish exile, it was the broadest in my collection. I knew him while living; he was, physically, a powerful man; his vital functions were manifested with great energy to the close of his life, which was by suicide; his brain had no perceptible depth; he lived out the last moment permitted by his organization; he was about 35 years old.

When the base of the brain is both broad and deep, the indication is a compound of the two conditions of high stimulus and high dynamis. This explains why some people of high stimulus live to be old. Of this compound constitution, General Scott is a fine example, and so was the notorious Aaron Burr. The late Professor Daniel Drake had but little of the high stimulus, and not more than a respectable portion of high dynamis, as he died at about the age of sixty-five years. The late Hon. John Randolph had less of the high stimulus condition than any other man I ever saw, but his dynamis sustained him to old age. A considerable portion of the high stimulus condition is indispensable to great action on the stage of life; and, hence, we find it to have been highly developed in Cæsar, Alexander, and Napoleon.

My cabinet investigations left me unable to doubt that longevity results from the downward development of the brain. Upon arriving at this conclusion, I directed my attention to society, and it soon became rumored that I had made a discovery by which I could tell how long a person would live. When this rumor reached the late Professor Evans, of the Ohio Medical College, he called on me and said:—"Professor Powell, I have learned that you have made a discovery by which you can tell how long a person will live; is it true, sir?" No, sir, I have not made such a discovery; but I have made one by which I

can tell whether a person will live a long or a short time; but even to this extent I am not confident, but am only so inclined to think. "Please to apply your discovery to me." Do you desire my candid opinion? "I do, sir." Then, sir, if I have made the discovery I think I have, you have but a short time to remain with us. "Oh! your discovery is all a humbug." What, if you please, is your definition of a humbug? "I did not mean that, but only you have not made the discovery you think you have." You are, possibly, correct, sir; for I have not arrived at a settled conclusion about it. The opinion I gave him proved to be correct, for in three years he was under the sod. I could present many very remarkable examples of my application of this discovery, but as they will avail nothing to my readers, I will pass on.

Early in this investigation, it became to me a question, whether this index of longevity decreased as age advanced or dissolution approached. From some observations, I was inclined to the affirmative, but, subsequently, some others inclined me to the negative; and, before I could settle the question, I became afflicted with hemiphlegia, which, in a great measure, arrested my observations. At the date of this discovery, my father was 78 years old, and his index of longevity was five-eighths of an inch; and, knowing him to have great tenacity of life, I was surprised to find his index so small, unless it had decreased, and only indicated the residue of his years. At all events, I resolved, if permitted, to watch it to the close of his life. On the 7th ult., he died at the age of 92 years, 1 month, and 20 days. I applied the line, and found no indication of longevity, which corresponded with the fact: he was dead; the line came down fully to the meatus. If I made no misapplication of the line, this case would be conclusive that this index decreases as age increases; having but one hand with which to manipulate, and the assistance of a child, I may, possibly have committed some error, though I repeated the measurement three times with the same result. But, before I conclude, finally, in the affirmative, I must have confirmation. Thus, I leave the question for others to settle.

Conceding that I have discovered the index of human longevity, the question arises, of what use is it to the medical profession? Not having had medical practice since making the discovery, my answer must consist of the evidence of those who have availed themselves of it in their practice. My observations, however, have forced upon me the conviction, that in the ratio of the index of longevity is the physiological power of the system to resist, contend with, and remove disease. Hence, it would be precedent for the physician to inform himself of the physiological ability of his patient to contend with disease; but more particularly should he have this knowledge before venturing a prognosis.

Nearly two years ago, Dr. Fox, of this city, now of California, made me a social call, and, in the course of conversation, he adverted to this discovery, saying:—"I obtained a knowledge of it from Dr. Fowler, of Cincinnati, who informed me that he acquired it personally from you; and such was his confidence in its verity that I was induced to give attention to it; and, upon becoming convinced of its truth, I resolved to make it available in my practice; and, for this purpose, I made a scale of a slip of a card and graduated into sixteenths of an inch, and attached one end of it to a line, so that when the line is adjusted, the scale depending over the meatus indicates the amount of the index. This little contrivance I always carry in my vest pocket. A few weeks since, I was called to see a sick child, about 4 years old. The case was phrenitis, and had been given up as irrecoverable by two physicians; and I found the case so hopeless that I resolved to do nothing without counsel, and sent to Cincinnati for a medical friend, in whose ability I had much confidence; but, while awaiting his arrival, I ascertained that the child's vital was six-eighths of an inch. As this was more than I had ever before seen in one so young, my inference was that he could not die but would rally and recover. My counsel arrived, examined the case, and said to me:—'We can do nothing in this case, the child will die.' 'No, sir,' said I, 'he will recover.' My friend rejoined:—'How can you think so when there is not even the fragment of a symptom in his favor?' 'I grant, sir, that, according to all the lights of the professsion, your opinion is correct; and yet, sir, it is my conviction that he will rally and recover.' 'Please to give me the data of your confidence.' 'When the case shall have terminated I will; but, as my data is such that you cannot appreciate, I beg to decline at present.' Suffice it to say, in a week the child was up and playing with the other children. When I informed my friend that the child recovered, he was greatly amazed and demanded of me my promise. He had heard of your discovery, but knew nothing about it. When called to cases of much violence, and find this index as small as one or two-eighths of an inch, I inform the friends, before I do anything, that the case will result fatally; and in no instance of the kind have I been disappointed. I think this discovery to be of great value to the physician."

In conclusion, I beg leave to introduce another witness. Something less than a year ago, a professional friend of the author made to him the following report:—"Ever since the announcement of your discovery of the index of human longevity I have directed my observation to it, and long ago reached the conclusion that it is a reality. A few months since, Professor ——— was required to extirpate an unusually large ovarian tumor, and solicited my assistance. It was granted; and, at the appointed time, I was with him; saw the lady; examined her index of viability, and found it to be as low as three-sixteenths of an inch; and, hence, I inferred that she would not, probably, live to recover from the operation, and so informed the Professor. 'Why,' said he, 'her physiological condition is very good.' I rejoined:—'That may be, but she has not physiological ability enough to recover from such a lesion as the extirpation of the tumor will require. He requested the data of my opinion. I gave it. He rejoined:—'Is not that Powell's pretended discovery?' 'It is, sir; but I do not hold Dr. Powell or his discovery responsible for my opinion. His discovery served to guide me; but I have made a knowledge of it as much my own as it is his; my opinion, therefore, is exclusively my own.' He operated, and did it well; and, for a time, the lady appeared to do as well as could have been expected under the most favorable circumstances,—but she did not live to recover from the operation.

Any person may in an hour become satisfied of the probable truth of this discovery, by comparing this index of those having a scrofulous diathesis with that of those who have a sound constitution.

When this index is as much as an inch, at or about adult age, there is a reasonable chance of life to four score years; when an inch and a-quarter, five score years, &c.

